# Application of Neon Ion Implantation to Generate Intermediate Energy Levels in the Band Gap of Boron-Doped Silicon as a Material for Photovoltaic Cells

**DOI:** 10.3390/ma14226950

**Published:** 2021-11-17

**Authors:** Paweł Węgierek, Justyna Pastuszak

**Affiliations:** Faculty of Electrical Engineering and Computer Science, Lublin University of Technology, Nadbystrzycka 38 A, 20-618 Lublin, Poland; j.pastuszak@pollub.pl

**Keywords:** intermediate band solar cells, ion implantation, photovoltaic cells efficiency, defects, electrical parameters of silicon, activation energy

## Abstract

The aim of the work is to present the possibility of generating intermediate levels in the band gap of p-type silicon doped with boron by using neon ion implantation in the aspect of improving the efficiency of photovoltaic cells made on its basis. The work contains an analysis of the influence of the dose of neon ions on the activation energy value of additional energy levels. The article presents the results of measurements of the capacitance and conductance of silicon samples with a resistivity of *ρ* = 0.4 Ω cm doped with boron, the structure of which was modified in the implantation process with Ne^+^ ions with the energy *E* = 100 keV and three different doses of *D* = 4.0 × 10^13^ cm^−2^, 2.2 × 10^14^ cm^−2^ and 4.0 × 10^14^ cm^−2^, respectively. Activation energies were determined on the basis of Arrhenius curves ln(e^t^(*T*_p_)/*T*_p_^2^) = f(1/k*T*_p_), where *T*_p_ is in the range from 200 K to 373 K and represents the sample temperature during the measurements, which were carried out for the frequencies *f*_p_ in the range from 1 kHz to 10 MHz. In the tested samples, additional energy levels were identified and their position in the semiconductor band gap was determined by estimating the activation energy value. The conducted analysis showed that by introducing appropriate defects in the silicon crystal lattice as a result of neon ion implantation with a specific dose and energy, it is possible to generate additional energy levels ∆*E* = 0.46 eV in the semiconductor band gap, the presence of which directly affects the efficiency of photovoltaic cells made on the basis of such a modified material.

## 1. Introduction

### 1.1. Background and Motivation

The dynamic development of the renewable energy sector, based primarily on photovoltaics [1,2,3] causes the need for research and development in a wide range of issues related to the solar energy conversion technology itself, production processes and operation, as well as sustainable development [4,5,6]. Particularly important is research aimed at increasing the efficiency of PV cells, which directly affects the profitability of their production manufacturing and application [3].

Currently, the mainstream development of photovoltaic technology concerns cells based on monocrystalline silicon, which constitute about 80% of all cells produced, while nearly 70% are based on p-type silicon, most often obtained by boron doping [7,8].

Analyzing the current state of the art in the field of research on methods of increasing the efficiency of crystalline silicon photovoltaic cells, a multi-threaded approach is required, focused on reducing internal losses that affect the efficiency of the cell. The total internal losses in a photovoltaic cell can be divided into electrical, optical and quantum losses [9]. Activities aimed at increasing the efficiency of electricity generation in solar cells are undertaken in all three areas, but the greatest development potential concerns methods of reducing optical and quantum losses, which are closely related to the internal structure and properties of the cell material. The key is the energy gap *E*_g_, which determines the energy a photon incident on the cell surface must have in order to be absorbed and participate in the photovoltaic conversion process. As it is known, for silicon, the value of *E*_g_ is equal to 1.12 eV; however, certain modifications may be introduced to its crystal lattice, as a result of which it is possible to change the physical properties of the material, in particular the distribution of energy levels, as well as the width of the energy gap. One of the methods enabling the modification of the internal structure of silicon, which results in the change of dielectric permittivity, resistivity and the creation of additional energy levels in the silicon band model, is ion implantation, which was shown in the works [10,11,12,13]. Moreover, it is known that the change of implantation parameters, such as energy and dose, as well as temperature and time of post-implantation annealing, allow for precise control of the process of introducing the above-mentioned structural modifications of the material.

### 1.2. Current Research Directions Using Ion Implantation

The results of mathematical modeling and laboratory experiments so far clearly indicate that in order to effectively reduce the internal optical losses it is necessary to be able to precisely control the distribution of crystal lattice defects in silicon cells. Such a possibility is provided by the use of ion implantation, therefore the idea of using defects to improve the absorption of silicon cells is the subject of many scientific works. The significant influence of defects on the absorption range of real silicon structures is discussed in the work [14]. It presents the results of optoelectronic characterization of p-type silicon layers with resistivities ranging from 5 Ω cm to 25 Ω cm, implanted at room temperature with sulfur, selenium, and tellurium ions with energies equal to 95 keV, 176 keV, and 245 keV, respectively, and doses ranging from 3 × 1014 cm^−2^ to 1016 cm^−2^. After implantation, the specimens were subjected to a pulsed laser melting (PLM) process. The transmission and reflection measurements showed a very high absorption coefficient in the energy range smaller than the silicon energy gap. The middle of the low-energy photon absorption band was located near the energy of 0.5 eV for sulfur and selenium ions and 0.3 eV for tellurium ions, while the extreme position of the lower edge of the band was determined to be within 0.05 eV. Nevertheless, it was observed that the bandwidth is not constant and increases with increasing dopant concentration. Moreover, annealing of strongly defected structures even at low temperatures of the order of (200 ÷ 400) °C leads to absorption weakening.

In order to explain the nature of this phenomenon and to identify the structures responsible for the existence of absorption in the low energy range, a model was developed for the transition of the defected silicon layer from the state of strong absorption to the state of weak absorption of photons with energies lower than the value of the energy gap. The basic assumptions of the model are presented in the work [15]. After approximation of the modeled process by the Arrhenius equation, the absorption activation energies for each type of implanted ions were obtained to be 0.338 eV, 0.471 eV, and 0.357 eV for sulfur, selenium, and tellurium ions, respectively. Based on this, several potential interstitial defects that may be responsible for the occurrence of the extended absorption phenomenon were identified.

Attempts to formulate a generalized description of the results obtained empirically, led to the development of a model based on which it was shown that the introduction of selected deep defects in the charge carrier capture region can be used to improve the efficiency of silicon solar cells. The course of the defect design process on the example of a silicon solar cell is presented in [16]. In particular, defects have been identified that facilitate the transport of majority charge carriers and/or prevent the accumulation of minority carriers, which impair recombination in the charge carrier capture region. Thus, the idea of deliberately introducing defects into the structure of a solar cell enables efficient transport of charge carriers and thus reduces optical losses.

Intensive research is also carried out to improve quantum losses in silicon cells. However, the most advanced concepts are currently in the laboratory verification phase, and no technical solution in this area has been commercialized. Nevertheless, taking into account the current state of the art in this area, as well as the research results presented in the world literature, it can be concluded that achieving this goal will be possible through the use of ion implantation. A research direction with a very high development potential in this field is the idea of IBSC cells (Intermediate Band Solar Cells). It is one of the third generation solar cell concepts and refers mainly to silicon. The theoretical basis of IBSC cells has been known for many years, and a model of an ideal cell of this type was presented in [17]. In this model, it was shown that the ideal efficiency of IBSC cells is equal to 63.2%, however, work is still ongoing to find a material that will allow for at least partial fulfillment of these assumptions.

The basic feature that distinguishes IBSC-type structures is an additional intermediate band, consisting of allowed energy states, located between the valence band and the conduction band. This configuration allows absorption of photons with energies lower than the energy gap through multiphase transport of charge carriers between valence band, intermediate band and conduction band. As a result, the ratio between the number of photons incident on the cell surface and the number of excited electrons is changed in favor of higher quantum efficiency, compared to standard silicon cells. At the same time, the photocurrent density of the cell is increased, and thus also its fill factor.

Despite the fact that from a theoretical point of view, the principle of operation of IBSC-type cells has been comprehensively described in [18], the problem of practical implementation of this concept in the form of a semiconductor material with an intermediate energy bandwidth, ensuring effective conversion of solar energy into electricity, still remains valid. Therefore, the mechanism of the formation of intermediate bands in semiconductors, as well as its utilitarian aspects, is the subject of many research papers, including a few selected current research directions based on the use of ion implantation [19,20,21,22,23,24,25]. One of them is an approach that includes the use of ion implantation to introduce dopants of very high concentration into the semiconductor substrate [25] or subjecting the silicon layer to implantation with metal ions of very high doses [20,23].

Successive progress in the use of titanium ions for this purpose was published in a series of theoretical and experimental papers [26,27,28]. At first, it was confirmed that implantation of a highly resistive silicon layer (*ρ* ≈ 200 Ω cm) with titanium ions with energies in the range of (35 ÷ 170) keV and doses in the range of (1015 ÷ 1016) cm^−2^ in selected cases causes a significant increase in the absorption of photons with energies below the energy gap value. Moreover, it has been shown that the strong photoconductivity is not related to the presence of crystal lattice defects, and the observed increase in the absorption coefficient and the extension of the lifetime of the photoexcited charge carriers are due to the high concentration of titanium ions [27]. As a result of further work, the formation of an intermediate energy band in a silicon layer implanted with titanium ions was confirmed at a sufficiently high dopant concentration [26]. Moreover, it was experimentally proven for the first time that recombination is inhibited when the concentration of titanium ions is high enough. The location of the center of the intermediate energy band was determined at a distance of (0.205 ÷ 0.278) eV below the conduction band, depending on the ion dose. After finding the existence of the intermediate band in the structure of the material, an attempt was made to characterize it experimentally. For this purpose, the position of deep energy levels forming the band was determined by thermal admittance spectroscopy. As a result, a clear relationship between the values of activation energy and active cross-section of charge carrier capture was observed. It was also confirmed that this relationship meets the Meyer–Neldel rule, which is characteristic of processes taking place in disordered structures, such as defective silicon, involving multiple excitation, which occurs in the case of emission and capture of charge carriers at deep energy levels [28]. The final result of the research work on the configuration of deep energy levels in silicon implanted with titanium ions is a comprehensive mathematical and physical model presented in [23], presenting in a consistent way conditions for the creation of additional energy levels with a different degree of density in the excited energy region. The model presents mechanisms for the formation of a continuous intermediate band, multiple discrete levels comprising a quasi-continuous band, or a single deep energy level, depending on the concentration of titanium dopants.

The presented analysis of the world literature on current research directions, confirms the effectiveness of the use of ion implantation to generate additional energy levels in the band structure of monocrystalline silicon, and the development potential in this field is so large that the conduct of further research work is justified and even necessary.

The research conducted by the authors of the article on the change of electrical parameters of silicon through the use of neon ion implantation [29,30,31,32] has led to the development of the author’s method for producing and identifying additional energy levels in the band structure of semiconductors. This direction fits perfectly into the current research trend and is in line with the market trends of increasing use of ion implantation in PV cell production [33,34].

The main goal of this paper is to determine the effect of Ne^+^ neon ion dose on the efficiency of generating additional energy levels in the band gap of p-type boron-doped silicon as a base material for photovoltaic cells. The research was aimed at determining the influence of the degree and type of the p-type silicon defect on its electrical parameters in terms of the possibility of producing intermediate energy levels in the semiconductor band gap.

## 2. Materials and Methods

Studies previously conducted by the authors’ team [13,32] allowed us to select the research material, which was p-type monocrystalline silicon commonly used in photovoltaics, doped with boron, with resistivity *ρ* = 0.4 Ω cm. The semiconductor wafers were implanted at room temperature with Ne^+^ neon ions with energy *E* = 100 keV and three different doses of *D*, 4.0 × 10^13^ cm^−2^, 2.2 × 10^14^ cm^−2^ and 4.0 × 10^14^ cm^−2^, respectively. In order to facilitate the identification of the experimental results presented in this paper, the wafers were labeled as follows:

- Si + B_1_—neon ion implantation with a dose *D* = 4.0 × 10^13^ cm^−2^,
- Si + B_2_—neon ion implantation with a dose *D* = 2.2 × 10^14^ cm^−2^,
- Si + B_3_—neon ion implantation with a dose *D* = 4.0 × 10^14^ cm^−2^.

Neon ions have been chosen due to the fact that they generate mainly point defects, whose deliberate introduction into the crystal lattice of silicon during the implantation process allows changing its basic electrical properties, such as energy gap width and resistivity. At the same time, it is well known that these parameters have a significant impact on the internal losses in photovoltaic cells [35].

In order to obtain data on determining the optimal dose of implanted neon ions due to the possibility of generating intermediate energy levels in the semiconductor’s band gap, experimental studies were carried out. These consisted of measuring the electrical properties of suitably prepared samples, such as capacitance and conductance at varying the frequency of the measurement signal and the instantaneous operating temperature of the test sample. The results of the measurements were recorded for operating temperatures in the range of 200 K to 373 K with a step of 10 K, after forcing in the circuit a signal of frequency in the range of 1 kHz to 10 MHz and a voltage of 0.5 V.

Between each measurement cycle, in order to remove unnecessary radiation defects, the samples were heat treated by 15-min isochronous annealing at temperatures *T*_a_ of 473 K, 598 K, 673 K, and 873 K. Based on the results of research work [36,37], the upper limit of annealing of the tested samples was set at 873 K. The thermal treatment of the samples at higher temperatures causes the complete disappearance of defects formed by ion implantation in the silicon structure, and thus eliminates the possibility of generating additional energy levels in the band gap.

**Figure 1:**
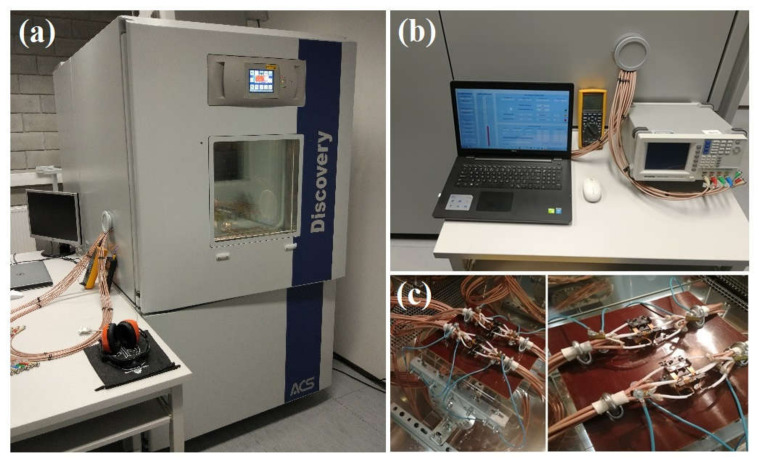
Laboratory stand for testing the electrical parameters of photovoltaic cells, (**a**)—Discovery DY600C environmental test chamber, (**b**)—measuring equipment (LCR meter, digital multimeters, computer software), (**c**)—measuring holders for placing silicon samples inside the climatic chamber.

In the course of the research work, a laboratory test stand was designed and constructed to test the electrical parameters of the samples described above. The construction of the stand was presented in detail in the work [36] and ensured repeatability of measurements due to the possibility of precise regulation and maintenance of conditions in which the tested object worked. The pictures of the research equipment, included in the laboratory stand, are shown in Figure 1.

The study was carried out using a Discovery DY600C climate chamber by the Italian Angelantoni Test Technologies company, using the proprietary PV Cells Meter computer program and the Winkratos software in the 5.00.04.00-STD-S7 version. The precision LCR-8000G Series LCR Meter by the Taiwanese GW Instek manufacturer was used to measure the capacitance and conductance values, while the sample temperature value was measured using Fluke 289 True-RMS Data Logging Multimeter by the American Fluke Corporation and Lutron TM-917 precision 0.01 degree thermometer by the American Lutron Electronics manufacturer [35,36].

The obtained measurement results allowed for the analysis of the effect of ion dose on the measured electrical parameters, i.e., capacitance and conductance, and consequently for the determination of the value of depositing additional energy levels in the band gap. For this purpose, the method of the Thermal Admittance Spectroscopy (TAS) was used which made it possible to determine the *e*^t^(*T*_p_) rate determining the thermal emission. Then, on the basis of the determined temperature dependence of the *e*^t^(*T*_p_) rate, as well as the Arrhenius curves, it was possible to calculate the value of the conduction activation energy Δ*E*, which determines the depth of the additional intermediate energy level in the silicon band gap.

The conducted research confirmed that the implantation of Ne^+^ neon ions causes the generation of radiation defects in the silicon crystal lattice, as a base material for photovoltaic cells, and gives the possibility to produce intermediate energy levels in the band gap, which can cause the absorption of photons with energies smaller than the width of the energy gap *E*_g_.

## 3. Results and Discussion

Measurements of the temperature dependence of the electrical properties of silicon, the methodology of which was presented in the previous section, provided information on the effects of deliberate changes in the crystal lattice structure by implantation of neon ions into the silicon substrate. The research task was to verify the influence of the dose of implanted ions on the value of activation energy of selected types of radiation defects, which are responsible for generation of additional energy levels in the band gap of p-type silicon, doped with boron. Taking into account the fact that the defects of silicon crystal lattice, formed as a result of neon ion implantation, can be used to reduce the quantum losses in solar cells by forming silicon structures meeting the assumptions of IBSC concepts, the solution of the task specified in this way imposed the necessity of detailed analysis of the configuration of energy levels existing in silicon subjected to neon ion implantation. Thermal Admittance Spectroscopy (TAS) was used to perform this analysis using an empirical method. At the same time, it should be noted that the method used by the authors differs fundamentally from the studies described in the introduction. The idea is to generate deep energy levels by intrinsic defects caused by implantation of electrophysically inert neon ions, and not to introduce additional defects into the silicon structure in the form of dopants and impurities from active elements. The mechanism of energy absorption and electric charge transfer in semiconductors implanted with neon ions is described in detail in [37].

The TAS technique allows the determination of the thermal emission rate *e*^t^, which is related to the temperature-induced flow of charge carriers over the potential barrier, which occurs as a result of the transfer of thermal energy to them in excess of the value of the output work. The value of the *e*^t^ rate for deep energy levels can be determined by measuring changes in capacitance *C* and conductance *G* as a function of temperature and frequency. The measured changes in *C* and *G* are the result of thermally induced fluctuations in the values of the time constants of emission and trapped charge carriers and are dependent on the frequency of the measurement signal. The measurement methodology assumes recording the values of *C* and *G* for specific frequencies of the measurement signal, under varying temperature conditions. Each deep energy level, occurring in the energy gap region of the semiconductor under study, exhibits a maximum of the conductance *G*_m_ at temperature *T*_m_ and an inflection point of the capacitance *C*_i_ at temperature *T*_i_, so that it is possible to determine the value of the thermal emission rate *e*^t^ using well-known formulas [38]:

(1) $$e^{t}\left(T_{m}\right)=\frac{\omega }{1.98},$$

(2) $$e^{t}\left(T_{i}\right)=\frac{\omega }{1.825},$$

where: *e*^t^(*T*_m_)—thermal emission rate at temperature *T*_m_, *e*^t^(*T*_i_)—thermal emission rate at temperature *T*_i_, *ω*—pulsation.

Therefore, performing measurements at different frequencies allows the temperature dependence of the *e*^t^ rate, from which the activation energy Δ*E* and the active cross-section of charge carrier capture by the trap *σ*_T_, which is a measure of the probability of free carrier capture by deep defect centers, can be determined.

The application of the Thermal Admittance Spectroscopy method allows the characterization of deep energy levels in those materials in which the dopant concentration is lower than the concentration determining the Mott transition limit. The reason for this situation is that in materials for which the dopant concentration exceeds the Mott limit, the Fermi level is located near the defect band. This, in turn, makes the samples resistive in nature, and thus there is no modulation of the depleted layer depth depending on the polarization voltage. In contrast, materials with dopant concentrations below the Mott limit show a strong dependence of the capacitance value on the polarization voltage, indicating the presence of depleted layer modulation [26].

For materials with dopant concentrations above the Mott transition limit, the temperature dependence of *C* and *G* does not show the existence of deep levels in the manner typical for dopant concentrations less than the Mott limit, i.e., through local maxima and inflection points. The lack of signs of the presence of deep energy levels in the admittance spectra is consistent with the assumptions of the intermediate energy band theory, which predicts that deep energy levels cease to be centers of recombination when an intermediate band is formed.

In the remainder of this paper, the analysis is limited to the results of conductance *G* measurements of the tested samples.

**Figure 2:**
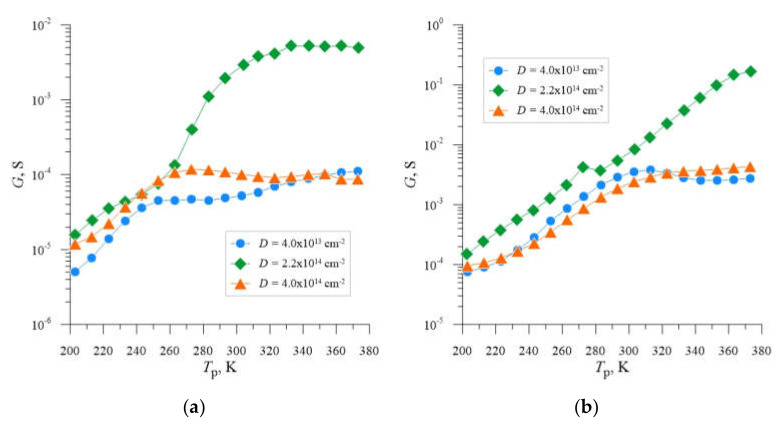
Dependencies *G* = f(*T*_p_) for silicon with resistivity *ρ* = 0.4 Ω cm, boron-doped, implanted with Ne^+^ neon ions with energy *E* = 100 keV, annealed at *T*_a_ = 598 K, determined for the frequency *f* of 10 kHz (**a**) and 1 MHz, respectively, (**b**) and three different doses of ions *D* = 4.0 × 10^13^ cm^−2^; 2.2 × 10^14^ cm^−2^; 4.0 × 10^14^ cm^−2^.

In the first phase of the analysis of the obtained results, the data on the temperature dependence of conductance for each implantation dose and selected frequencies were collected and presented in graphical form in Figure 2a,b. The analysis of the presented dependencies indicates that in the case of samples subjected to ion implantation with each of the applied doses changes were caused in conductance, which are associated with the phenomena of charging and discharging of deep energy levels, taking into account the frequency of the measurement signal. The occurrence of deep energy levels in the energy gap region of the investigated semiconductor samples is confirmed by the occurrence of inflection points and local maxima in the courses of conductance dependence as a function of temperature. Taking this into account, it can be noted that in the case of all investigated samples such phenomena do not occur. Moreover, it can be observed that the amplitudes of the local maxima of conductance measured for individual samples reach different values, which indicates different concentration of deep recombination centers, the highest in the case of the sample implanted with a dose of *D* = 2.2 × 10^14^ cm*^−^*^2^.

**Figure 3:**
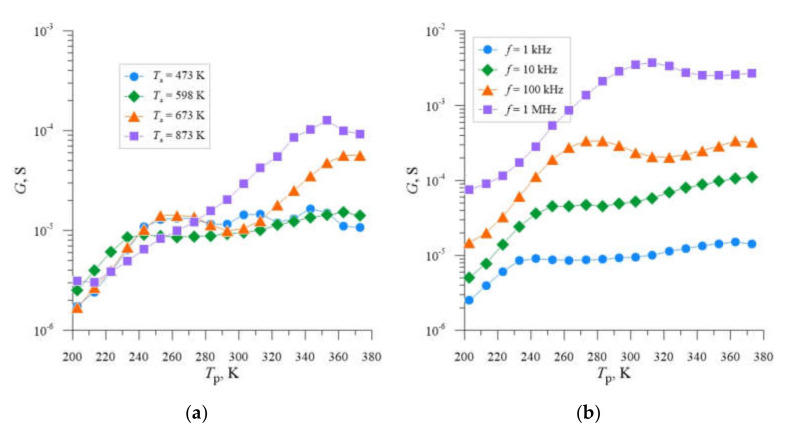
Dependencies *G* = f(*T*_p_) for silicon with resistivity *ρ* = 0.4 Ω cm, boron-doped, implanted with Ne^+^ neon ions with energy *E* = 100 keV and dose *D* = 4.0 × 10^13^ cm^−2^, determined for the frequency *f* = 1 kHz and four different annealing temperatures *T*_a_ (**a**) and annealing temperature *T*_a_ = 598 K and four different measurement frequencies *f* (**b**).

**Figure 4:**
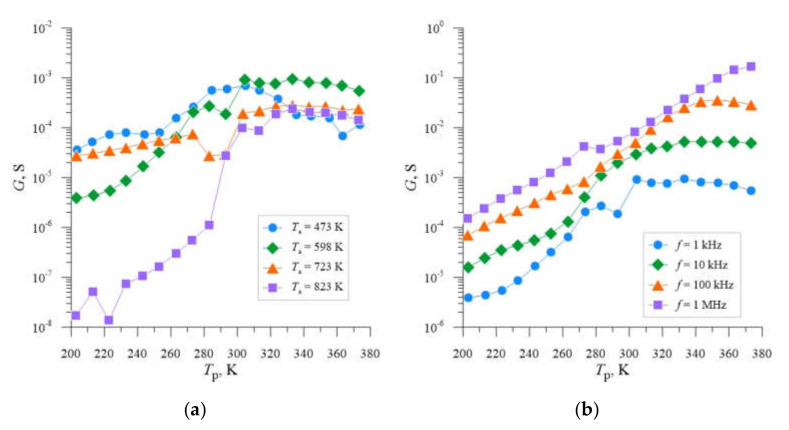
Dependencies *G* = f(*T*_p_) for silicon with resistivity *ρ* = 0.4 Ω cm, boron-doped, implanted with Ne^+^ neon ions with energy *E* = 100 keV and dose *D* = 2.2 × 10^14^ cm^−2^, determined for the frequency *f* = 1 kHz and four different annealing temperatures *T*_a_ (**a**) and annealing temperature *T*_a_ = 598 K and four different measurement frequencies *f* (**b**).

**Figure 5:**
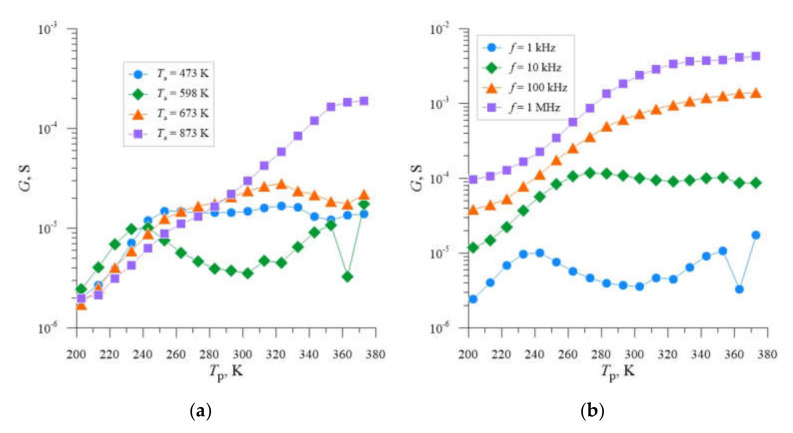
Dependencies *G* = f(*T*_p_) for silicon with resistivity *ρ* = 0.4 Ω cm, boron-doped, implanted with Ne^+^ neon ions with energy *E* = 100 keV and dose *D* = 4.0 × 10^14^ cm^−2^, determined for the frequency *f* = 1 kHz and four different annealing temperatures *T*_a_ (**a**) and annealing temperature *T*_a_ = 598 K and four different measurement frequencies *f* (**b**).

The choice of the post-implantation annealing temperature *T*_a_ = 598 K as the optimum temperature due to the selection of Si-B3-type radiation defects with the highest concentration, which introduce additional energy levels in the semiconductor’s band gap, follows from the analysis of the literature [39] and previous studies [40]. In order to confirm this fact, the *G* = f(*T*_p_) dependencies were developed for individual samples and four different annealing temperatures *T*_a_ (Figure 3a, Figure 4a and Figure 5a), from which it can be seen that for each of the implantation doses used, local maxima and characteristic inflection of the *G* = f(*T*_p_) curve occur for temperature *T*_a_ = 598 K. Figure 3b, Figure 4b and Figure 5b show analogous *G* = f(*T*_p_) relationships for individual samples, for the previously selected post-implantation annealing temperature *T*_a_ = 598 K and different frequencies *f*. In this case, we also observe local conductance maxima whose location shifts towards higher sample temperatures *T*_p_ with increasing frequency.

**Figure 6:**
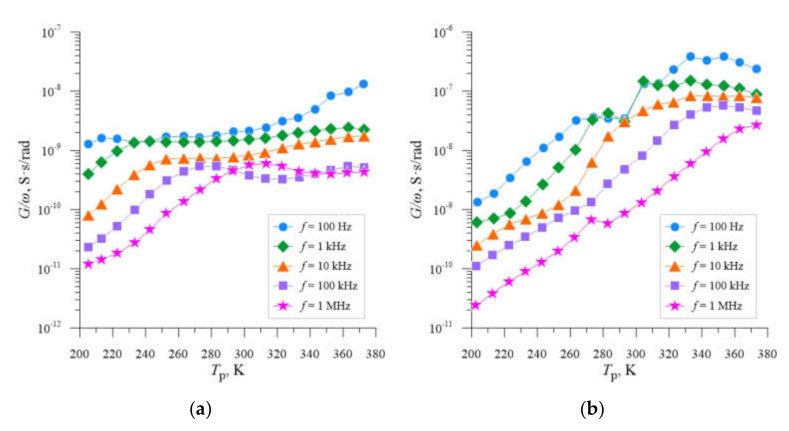

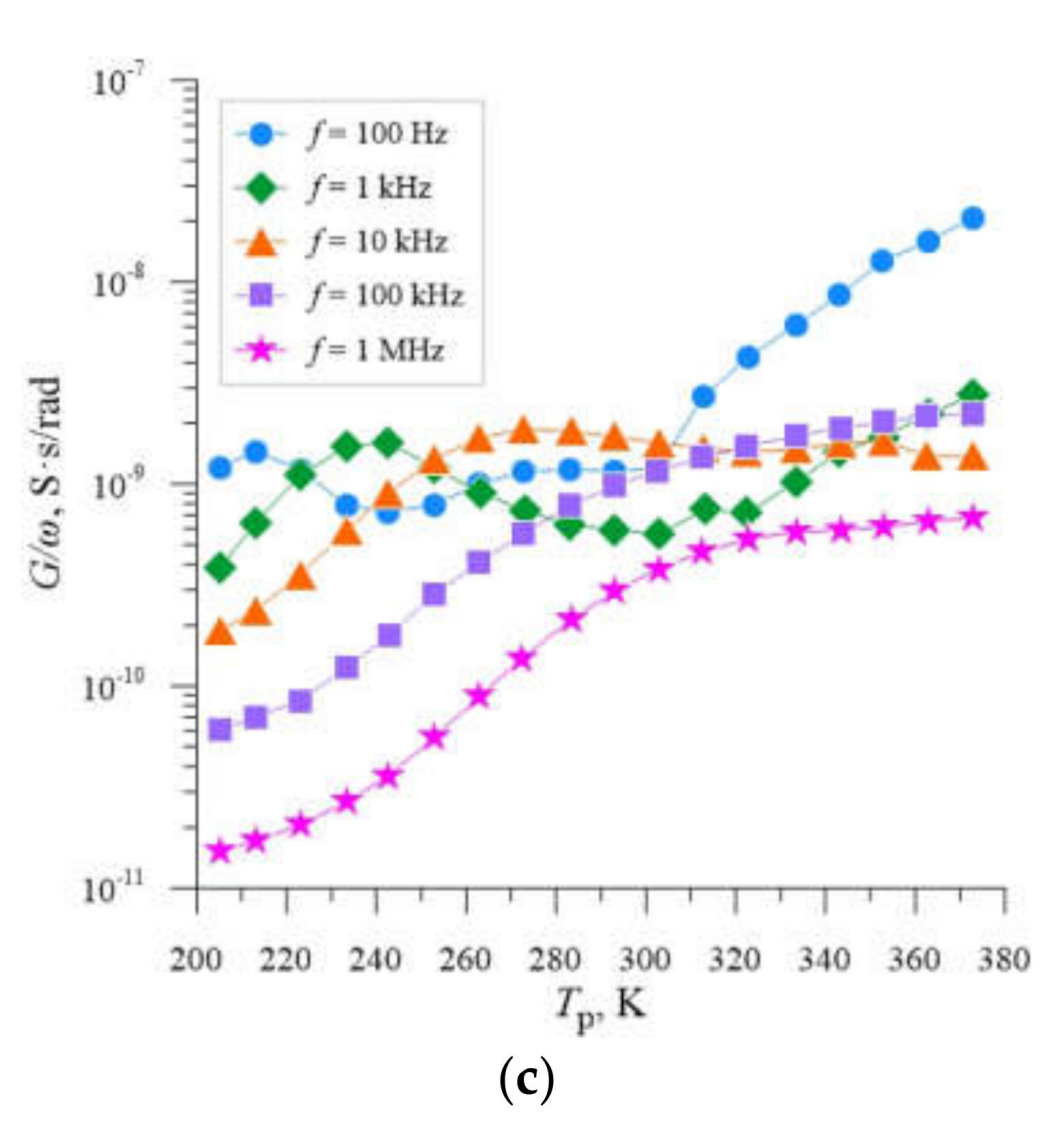
Dependencies *G*/*ω* = f(*T*_p_) for silicon with resistivity *ρ* = 0.4 Ω cm, boron-doped, implanted with Ne^+^ neon ions with energy *E* = 100 keV and doses of *D* = 4.0 × 10^13^ cm^−2^ (**a**), *D* = 2.2 × 10^14^ cm^−2^ (**b**), *D* = 4.0 × 10^14^ cm^−2^, respectively, (**c**) determined for different *f* and the annealing temperature *T*_a_ = 598 K.

The second phase of the study was to determine the value of activation energy of deep levels found in the tested samples. For this part of the analysis, in accordance with the justification presented above, the samples annealed at *T*_a_ = 598 K were chosen, for which the doses of neon ions were 4.0 × 10^13^ cm^−2^, 2.2 × 10^14^ cm^−2^ and 4.0 × 10^14^ cm^−2^. The calculations were performed for the voltage of measuring signal *U*_p_ = 0.5 V. In all cases, the analysis procedure was analogous. It included the determination of the dependence of the measured conductance *G*, related to the value of the pulsation *ω* as a function of the measurement temperature *T*_p_ for different values of the frequency of the measurement signal *f*, as shown in Figure 6.

**Figure 7:**
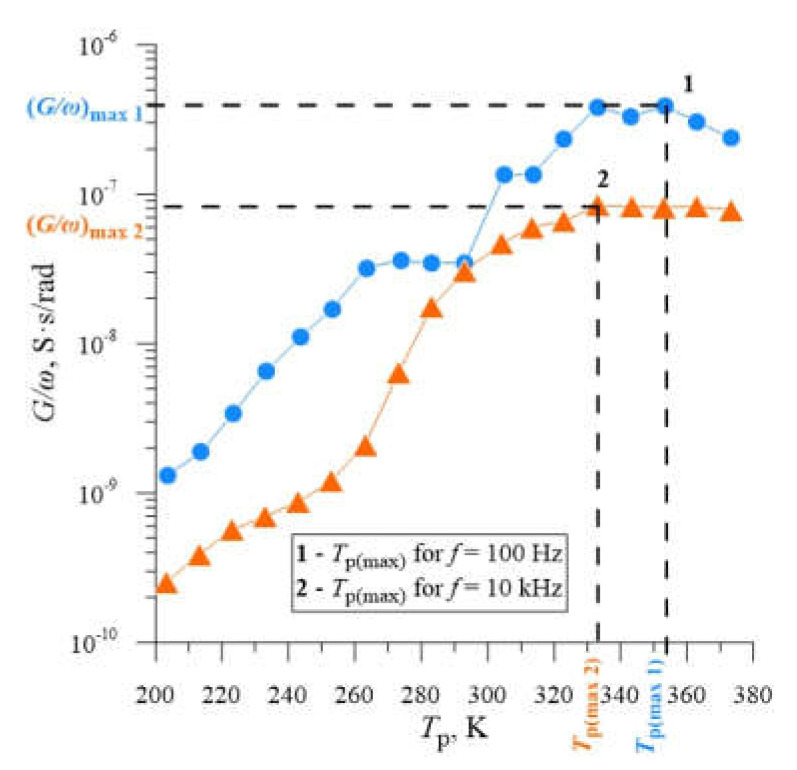
The method of determining the value of *G*_max_/*ω* and *T*_p(max)_ on the example of the dependence *G*/*ω* = f(*T*_p_) for silicon with resistivity *ρ* = 0.4 Ω cm, doped with boron, implanted with neon ions Ne^+^ with energy *E* = 100 keV and dose *D* = 2.2 × 10^14^ cm^−2^, plotted for the measurement frequencies *f* = 100 Hz and 10 kHz and the annealing temperature *T*_a_ = 598 K.

As can be seen, for each plotted relation, the location of the local maxima is characteristic of a certain frequency *f* and thus of the corresponding pulsation, and changes with increasing the operating temperature of the sample *T*_p_. According to the explanation suggested in the work [41], the local maximum of the conductance *G*_max_, which corresponds to the value of *G*_max_/*ω*, determined for the temperature value *T*_p(max)_, as shown in Figure 7, is related to the thermal emission rate of the deep energy level *e*^t^(*T*_p(max)_). By calculating the values of the pulsation *ω*, for each frequency, we can calculate the values of *e*^t^(*T*_p(max)_), according to Equation (1).

On the other hand, it is known that the thermal emission rate of the deep energy level *e*^t^(*T*) can be expressed as follows [38]:

(3) $$e^{t}\left(T\right)=\sigma_{T}\nu_{\mathrm{th}}N_{c}\exp\left(-\frac{∆E}{kT}\right),$$

where: *σ*_T_—active carrier capture cross section, *ν*_th_—thermal rate of charge carriers, *N*_C_—concentration of defects in conduction band, Δ*E*—activation energy, *T*—temperature, *k*—the Boltzmann’s constant.

Since it is known that the value of *ν*_th_ is proportional to *T*^1/2^, while the value of *N*_C_ is proportional to *T*^3/2^, Equation (3) can be transformed to the following form:

(4) $$e^{t}\left(T\right)/T^{2}=\gamma \sigma_{T}\exp\left(-\frac{∆E}{kT}\right),$$

where *γ* includes the value of the coefficients *ν*_th_ and *N*_C_ and does not depend on temperature

**Figure 8:**
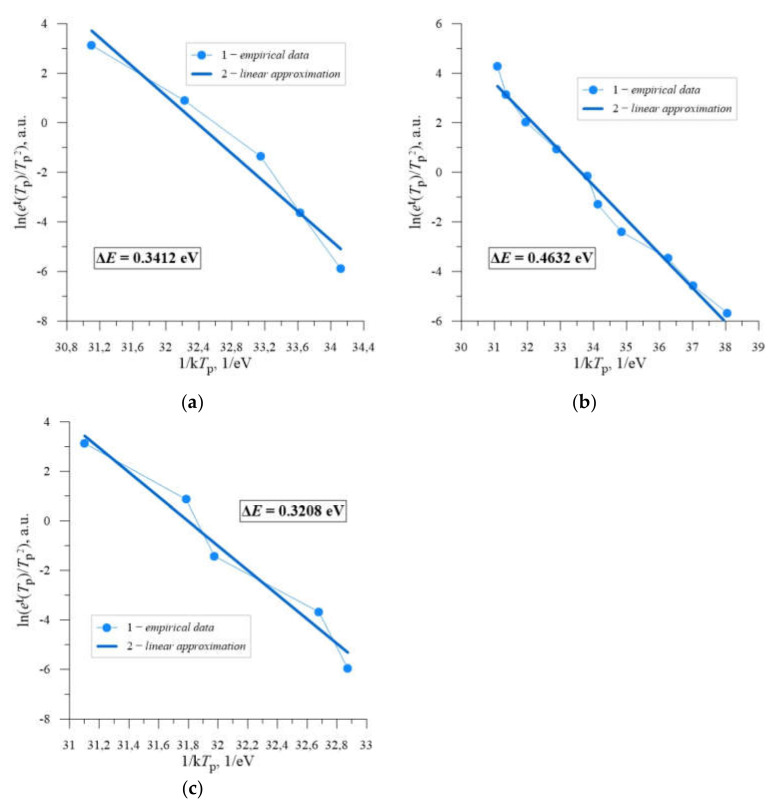
The Arrhenius plots of the function ln(*e*^t^(*T*_p_)/*T*_p_^2^) = f(1/*kT*_p_) for silicon with *ρ* = 0.4 Ω cm, doped with boron, implanted with Ne^+^ neon ions with energy *E* = 100 keV and doses *D* = 4.0 × 10^14^ cm^−2^ (**a**), *D* = 2.2 × 10^14^ cm^−2^ (**b**) and *D* = 4.0 × 10^14^ cm^−2^ (**c**), annealed at *T*_a_ = 598 K: 1—empirical data, 2—linear approximation.

The relation (4) is in the form of Arrhenius equation, so it is possible to determine the activation energy of deep energy levels by approximation of experimental data with a linear function. The results of such approximation for an annealing temperature of 598 K are presented in Figure 8.

On the basis of the analysis carried out, the existence of intermediate energy levels in the structure of the studied samples in the semiconductor band gap, with activation energies dependent on the dose of implanted neon ions, amounting to respectively:

- sample Si + B_1_—Δ*E*_1_= 0.3412 eV for *D* = 4.0 × 10^13^ cm^−2^ (Figure 8a),
- sample Si + B_2_—Δ*E*_2_ = 0.4632 eV for *D* = 2.2 × 10^14^ cm^−2^ (Figure 8b),
- sample Si + B_3_—Δ*E*_3_ = 0.3208 eV dla *D* = 4.0 × 10^14^ cm^−2^ (Figure 8c).

## 4. Conclusions

The analysis performed was focused on the identification of intermediate energy levels in the band gap of boron-doped silicon with resistivity *ρ* = 0.4 Ω cm, resulting from implantation with Ne^+^ ions of different doses. For each of the three applied ion doses, which are 4.0 × 10^13^ cm^−2^, 2.2 × 10^14^ cm^−2^ and 4.0 × 10^14^ cm^−2^, the existence of deep energy levels with activation energies of 0.34 eV, 0.46 eV and 0.32 eV, respectively, was observed.

Thus, the possibility of using the implantation of neon ions to defect silicon structures in such a way that additional energy levels are generated in the band gap of the semiconductor was confirmed. The results of the conducted research should be considered in terms of potential use in the manufacturing process, by the method of ion implantation, of monocrystalline silicon photovoltaic cells. Especially noteworthy are the results obtained with the implantation of neon ions at a dose of 2.2 × 10^14^ cm^−2^, which allowed the generation of an additional energy level with an activation energy of Δ*E* = 0.46 eV, i.e., near the center of the silicon band gap (*E*_g_ = 1.12 eV). This will provide a multi-step mechanism for the absorption of photons with energies below the width of the band gap.

The practical implementation of the concept of energy-efficient silicon photovoltaic cells with intermediate energy levels in the semiconductor band gap, generated by means of neon ion implantation, requires further research. They must be focused on the development of a technology for manufacturing complete silicon cells, taking into account the additional process of implantation of neon ions and the search for optimal implantation parameters, i.e., energy and ion dose, adapted to the properties of the substrate material, including primarily the type of dopant and resistivity.

## Data Availability

Data sharing is not applicable for this article.
